# Influence of Coating Application Methods on the Postharvest Quality of Cassava

**DOI:** 10.1155/2019/2148914

**Published:** 2019-02-04

**Authors:** Loreto Atieno, Willis Owino, Elijah M. Ateka, Jane Ambuko

**Affiliations:** ^1^Department of Food Science and Technology, Jomo Kenyatta University of Agriculture and Technology, P.O. Box 62000-00200, Nairobi, Kenya; ^2^Department of Horticulture, Jomo Kenyatta University of Agriculture and Technology, P.O. Box 62000-00200, Nairobi, Kenya; ^3^Department of Plant Science and Crop Protection, University of Nairobi, P.O. Box 29053, Nairobi, Kenya

## Abstract

Various modes of edible coating application vary in their coat dispersion and film formation, hence the need to determine the most effective mode of application for cassava. Edible surface coatings have been found to be effective in preserving the quality of various food products. However, there are variations in effectiveness among the different coating solutions, hence the need for optimization of the concentrations of the gums used. This study aimed at determining the most efficient coating application method on the cassava postharvest quality. Physiologically mature cassava (variety KME 1) was harvested and divided into seven portions. The various portions were coated using 1.5% xanthan gum, 1.5% xanthan/guar gum, and 2% xanthan/guar gum by both dipping and spraying method. There was no significant difference on the colour, total cyanide, ethylene production, and total phenolic content between the two application methods. The 2% xanthan/guar gum coating showed a significant difference on the dry matter content while the 1.5% xanthan gum coating had a significant difference on the respiration rate and weight loss. The 1.5 xanthan treated roots had a final dry matter content of 72.5% for the sprayed samples and 75.98% for the dipped sample while the 2% xanthan/guar gum treated roots had a final dry matter content of 64.6% and 74.1% for the dipped and sprayed root samples, respectively. The 1.5% xanthan and 2% xanthan/guar gum treated roots showed no significant difference in their action on dry matter content. The 1.5% xanthan/guar dipped and sprayed samples differed significantly on their effect on flesh firmness with final values of 35.4N and 46.1N, respectively, at 20 days after harvest. This study suggested that based on the coating solution and the parameters being observed, there generally was no varying effect of dipping and spraying methods of coating application. The choice of the efficient mode of application to use will depend on other factors such as the easiness of application.

## 1. Introduction

Cassava is widely consumed in the tropical regions [1]. The total cassava production in Africa increased by 18.9% from 132,200,764 tons to 157,271,697 tons in the period of the year 2010 to 2016 [2]. Despite this increase in production, the cassava suffers a physiological disorder that occurs after 24-72 hours and this renders it unpalatable [3]. Postharvest physiological deterioration (PPD) is associated with mechanical damage that occurs during the root harvesting process as the root is separated from the plant creating a wound [4]. Since this wounding is unavoidable various techniques may be employed to delay PPD occurrence. Edible coatings have long been used to retain the shelf life of various commodities while still retaining their quality by formation of a thin edible film [5]. Edible films are thin layers formed on a food product that has been coated. These products have been used for a long time to prevent loss of moisture from the product, reduce ethylene and respiration rates, and lead to an eventual extension in the shelf life of the product [6]. The edible films can be consumed with the product as they are generally regarded as safe (GRAS) and they do not impart any extraneous flavours on the product [7]. They have also been found to have antibrowning effects, for instance, in cucumbers [8].

Coating of food products can be done by either dipping, spraying, brushing, extrusion, panning, or solvent casting [9]. The mostly used modes of application are dipping and spraying due to their high convenience [10]. Dipping is one of the oldest used coating techniques from as early as the 12th century. It has a very simplified mode of application in which the product is dipped into the coating solution for a specified amount of time, drained, and left to dry before storage [10]. It can lead to formation of thick layers on the solid foods which may cause problems during storage due to anaerobic respiration [11]. This mode of application may lead to dilution of the coating solution leading to unwanted residue on the product [12]. It is also difficult to get good adhesion of the coating solution on the product due to the draining effect of the solution; hence multiple dipping may be necessary to ensure full coverage on the product [7]. It is best used for irregularly shaped products.

The spraying technology is mostly used in food industries due to its convenience [10]. This technique uses a spray machine in which the coating solution is forced out of the nozzle onto the surface of the produce [7]. It is mostly preferred when using less viscous solutions as the highly viscous solutions are not easily sprayed and they also block the nozzles. The spraying efficiency depends on the nozzle size, coating fluidity, and the amount of pressure put to release the fluid through the nozzles [11]. This mode of application leads to formation of an even coat due to the similarity in the drop size distribution and similar overlap effect [13]. It is mostly used on products with a large surface area [7].

The effectiveness of the two modes of application differs based on the food products being coated and there is contradicting information on the most efficient between the two methods. Moreover, gums have been found to be an excellent moisture loss barrier and good in freshness preservation [14], but the different fruits and vegetables react differently to various edible coatings. Therefore, this study was carried out with the objective of determining the best gums and technique for coating (dipping versus spraying) cassava roots that could lead to optimal shelf life extension with minimal or no effects on its nutritional quality.

## 2. Materials and Methods

### 2.1. Acquisition of Raw Materials

In the preliminary study, xanthan and guar gum were sourced from Sigma-Aldrich. The preliminary experiments indicated that the effectiveness of the gums sourced from Sigma-Aldrich was similar to the food grade gums sourced from a food ingredient supplier in Nairobi. Hence for the main experiment, the gums were sourced from a local food ingredient supplier. Fresh cassava root (*Manihot esculenta*) crops of variety KME 1 at physiological maturity were obtained from the JKUAT farm. The cassava roots were transported to the JKUAT postharvest laboratory and sorted according to size (50-60 cm long) and the amount of injuries. They were then cleaned using a soft brush to avoid bruising.

### 2.2. Research Design

Completely Randomized Design was used for the experiment. The treatments included 3 different concentrations of 1.5% xanthan gum, 1.5% xanthan/guar gum, and 2% xanthan/guar gum. The different treatments were applied by both dipping and spraying method. The data was recorded at the fresh stage and at 2 day intervals for a storage duration of twenty days.

### 2.3. Preparation and Application of Coating Formulation

Two different coating application methods were tested to determine the most effective mode of application to be used on the cassava roots. Coating was performed on the same day that the cassava roots were harvested. The differently treated roots were then subjected to physical, physiological, and chemical analysis for the entire storage duration at two-day intervals.

The coating formulations used were 1.5% xanthan gum, 1.5% xanthan/guar gum, and 2% xanthan/guar gum. The 1.5% xanthan gum was prepared by dispensing 1.5 g of xanthan gum into 100 ml of distilled water. This was heated at 40°C on a magnetic stirrer for one hour. The 1.5% xanthan/guar gum solution was prepared by dispensing 0.75 grams of xanthan gum and 0.75 grams of guar gum into 100 ml of distilled water while the 2% xanthan guar gum solution was prepared by dispensing 1 gram of both xanthan gum and guar gum into 100 ml of distilled water and heated at 60°C on a magnetic stirrer for one hour. Coating was done on the same day that the roots were harvested. For the dipping method of application, the already cleaned roots were immersed into a bucket containing the coating solution. The roots were let to stay in the solution for three minutes after which they were removed and excess solution was left to drip off the cassava for one minute. The roots were then placed in clean plastic crates and left to dry after which they were stored for the twenty day storage duration. For the spraying technique, the solution was put in a hand held sprayer and this was then dispersed onto the cleaned roots as demonstrated by Pérez-Gallardo et al. [15]. The roots were then placed in clean crates for air drying as recorded by Dhall [14]. This was done in the open air. Drying took 12 hours. Storage was done at 25°C. Cassava roots under the different treatments were then tested for the twenty days storage duration at two-day intervals to determine the effect of the coatings on the physical, physiological, and chemical properties of the cassava roots.

### 2.4. Determination of Flesh Firmness

A hand held penetrometer (N/g model ver 0.2, CRD-100D, Sun Scientific Co., Ltd, Japan) fitted with a probe was used to determine the firmness of flesh of the roots to a depth of 10 mm and the corresponding force required to penetrate this depth was determined according to Famiani et al. [16]. A cylindrical cork borer was used to get even samples of 2 cm length. The diameter of the cork borer was 10 mm. The test was carried out at a probe speed of 6 mm/s. The speed set on the hand held penetrometer. The firmness of each cassava was measured at three points along the equatorial region of the cassava. Firmness was taken to be the resistance of the flesh to the penetration of the plunger expressed as mean force in Newtons.

### 2.5. Determination of Colour Change

The colour of the cassava samples (3 replicates per treatment) was determined using a hunter lab colour difference meter (Minolta, Tokyo, Japan) according to Hernández-Muñoz et al. [17]. The instrument was standardized each time with a white ceramic plate. The colour was measured at four different regions along the midsection spaced 90°C apart. Results were tabulated and the L*∗* values used to determine the rate of color changes of the flesh with time.

### 2.6. Determination of Weight Loss

Cassava samples (3 replicates per treatment) were weighed while fresh and at an interval of two days for twenty days. The difference between initial and final root weight was determined for that storage period and expressed as a percentage on a fresh weight basis [18].

(1) %  weight  loss=Initial  weight  of  sample−current  weight  of  sampleInitial  weight  of  sample∗100

### 2.7. Determination of Dry Matter Content

This was determined according to Ebah-Djedji et al. [19]. 20 g of the chopped and ground roots was oven-dried at 105°C for 24 hours. Dry matter was then expressed as a percentage of the dry weight relative to the fresh weight.

(2) %  dry  matter  content=100−Dish  and  Sample  weight−Dish  weightSample  weight∗100

### 2.8. Determination of Total Phenolic Content

The amount of total phenolic contents was determined by the Folin-Ciocalteu method as described by Ainsworth and Gillespie [20] with modifications. Two grams (2 g) of the cassava root was ground in an ice-cold mortar and pestle using 20 ml of ice-cold 95% methanol. The samples were then vortexed and incubated at 25°C for 72 hours in the dark. The puree was then filtered to remove debris, the residue centrifuged at 13,000g for 10 minutes at room temperature, and the supernatant collected. The sample was then passed through a 0.45*μ*l membrane filter. To 1 ml of the sample extract and the standard, 2 ml of 10% (vol/vol) Folin-Ciocalteu was added and vortexed and 4 ml of saturated Na_2_CO_3_ solution was then added. The mixture was then allowed to stand at 25°C for 2 hours and the absorbance measured at 765 nm using UV-vis spectrophotometer. A standard curve was generated using the absorbances of gallic acid as standards in ppm. The amount of total phenols was expressed as gallic acid equivalents per 100 g of the sample.

### 2.9. Determination of Cyanide Content

Total HCN was analyzed using the alkaline titration method according to Famurewa and Emuekele [21]. Approximately 4 g of the cassava was ground and passed through a sieve. This was then soaked in a mixture of distilled water (40ml) and orthophosphoric acid. The samples were then thoroughly mixed and left to stand at room temperature overnight for 24 hours. This was done to set free the hydrocyanic acid. The remaining sample was then transferred into a distillation flask and a drop of paraffin was added to the broken chips to act as an antifoaming agent. The sample was then distilled and about 45 ml of the distillate was collected in the receiving flask containing 4 ml distilled water and 0.1g of sodium hydroxide pellets. The distillate was then transferred to a 50 ml volumetric flask and made up to the mark using distilled water. 1.6ml of 5% potassium iodide was then added and titrated against 0.01M Ag(NO_3_). The endpoint was indicated by a faint but permanent turbidity. The total HCN content in mg/kg was calculated as total

(3) $$\begin{array}{c} \text{HCN content}=\frac{13.5*TV}{M} \end{array}$$

where

  TV=titre value and
  M=mass of sample.

### 2.10. Determination of Respiration Rate

Air tight containers of specific known volume fitted with self-sealing rubber septums were used. The weight of each cassava root was measured. The samples were then incubated in the air-tight plastic containers for one hour. After one hour, 1 ml of the headspace gas was drawn from each container using an air-tight syringe and injected into a gas chromatography (Shimadzu Corp., Kyoto, Japan, model GC-8A) fitted with a thermal conductivity detector and a Propak N column. The respiration rate was measured as mg CO2 per Kg per hour.

### 2.11. Determination of Ethylene Production Rate

This was done according to Fugate et al. [22] with a few modifications. Air tight containers of specific known volume fitted with self-sealing rubber septums were used. The weight of each cassava root to be used was taken. The samples were then incubated in the air-tight plastic containers. After one hour, 1 ml of the headspace gas was drawn from each container using an air-tight syringe and injected into a gas chromatography model GC-9A, Shimadzu Corp., Kyoto, Japan. The detector used was the flame ionization detector fitted with activated alumina. Standard curves were generated by injecting pure gas samples of known concentrations. The rate of ethylene production was then calculated in nl C_2_H_4_/g/h.

### 2.12. Statistical Analysis

Comparisons among the various treatments and storage duration effects were determined by analysis of variance (ANOVA) while mean separations were performed using Tukey test at *α*=0.05 significance level. Data analysis was carried out using Genstat statistical package 12th edition.

## 3. Results and Discussion

### 3.1. Flesh Firmness

The flesh firmness of the treated samples was determined and recorded from the first day after harvest to 20 days after harvest (DAH) as shown in Table 1.

The 1.5% xanthan treated samples were both dipped and sprayed with the coating solution. The dipped sample attained a flesh firmness peak of 91.4N at 12DAH while the sprayed sample attained its firmness peak of 95.6N on the same day. From 12DAH onwards, the two samples declined in their flesh firmness Newtons as they approached 20DAH. At this point, the dipped sample had 21.2N while the sprayed sample had a slightly higher flesh firmness of 29.1N with no significant (P>0.05) differences.

In the 1.5% xanthan/guar treated cassava root, the dipped sample attained its peak of 95.8N at 8DAH while the sprayed samples attained its peak of 95.0N at 10DAH. Thereafter, there was a general decline in the flesh firmness of the treated samples as they approached 20DAH. By 20DAH, the sprayed sample had a higher flesh firmness of 46.1N while the dipped sample had 35.4N. At 20DAH, the dipped and sprayed sample showed a significant (P≤0.05) difference on the flesh firmness of the treated samples.

The 2% xanthan/guar treated roots attained firmness peak of 88.0N for sprayed samples at 8DAH while the dipped sample attained the peak of 97.2N at 10DAH. This was followed by a decline in the flesh firmness of the treated roots. The dipped sample had a final firmness of 24.5N while the sprayed sample had a firmness of 24.3N at 20DAH. Upon coating, the treated samples generally showed an increase in the flesh firmness to 10DAH followed by a decrease till 20DAH as shown in Table 1.

At 20DAH, the control sample had a flesh firmness of 4.0N which was significantly (P>0.05) different from all the treated cassava root samples. There was generally no significant differences of the two application methods on the flesh firmness.

The change in flesh firmness of the cassava root samples is affected by the occurrence and development of the PPD. After harvesting of the cassava root crop, there is an increase in the flesh firmness of the cassava and then a later decline in the firmness. The permeabilizing of the cell membrane enhances the loss of the water and this may have led to the increase in flesh firmness [23]. With the progress of the PPD, there is softening of the root and eventual decay [1]. This may have led to the decline of the flesh firmness of the root. The coating process reduced the rate at which the flesh firmness changed. As the root storage progressed towards 20DAH, the control roots started decaying leading to a final flesh firmness of 4.0N. This may have been due to the effects of the PPD that lead to microbial decay.

### 3.2. Colour

The cassava root crop suffers cell disruption during the harvesting process and other subsequent postharvest activities such as transportation, sorting and washing. This cell disruption leads to PPD development. With the onset of PPD, there is formation of blue/black streaks on the vascular bundles of the cassava. The L*∗* values recorded during colour evaluation of the cassava root crop range from black (0) to white (100). A reduction in the L*∗* values from the day of harvest means that there is darkening of the cassava flesh [24]. 1.5% xanthan treated samples showed a decline in the L*∗* value from the first day after coating. At 20 DAH, the dipped sample had no significant (P>0.05) difference from the sprayed sample though it had a higher L*∗* value of 79.5 while the sprayed sample had 65.9.

There also was a decline in the L*∗* value of the 1.5 xanthan/guar treated samples. Upon coating, the L*∗* value was 92.4 and this decreased for both the dipped and the sprayed samples. At 20DAH, the dipped sample had a higher L*∗* value of 77.1 hence a whiter flesh than the sprayed sample that had 60.2. This was significantly (P≤0.05) different for the two samples.

The 2% xanthan/guar gum treated root samples had a similar declining trend in the L*∗* value of the flesh colour. However, the sprayed samples showed a better value as compared to the dipped samples at 20DAH. At 20DAH, the sprayed sample had an L*∗* value of 83.2 while the dipped sample had 67.90 which was significantly (P≤0.05) different from all the treated samples at this day (Table 2).

There were generally no significant (P>0.05) differences among the treatments that were dipped and those that were sprayed with the coating solutions. This suggested that the two different coating applications had the same activity in terms of maintenance of the cassava root flesh colour.

After harvest of the cassava root crops, there is development of PPD that affected the colour of the flesh as recorded by Liu [25]. The colour of a fresh sweet variety cassava root was white as recorded by Ademosun et al. [26], but with the development of PPD, it developed blue black streaks. This initial L*∗* value of the fresh sample was recorded as 92.43 and, with its deterioration, the value decreased towards zero. This change in colour was manifested by blue/black streaking of the xylem bundles [1]. The control sample had a drastic change in colour as compared to the coated samples and this may have been due to the different efficacy levels of the coating solutions.

### 3.3. Weight Loss

The weight loss of the treated root samples was determined and recorded from the first day to 20DAH. There was an increase in the percentage weight loss of the various samples till 20DAH

The 1.5% xanthan treated root samples showed an increase in weight loss to 30.0% for the dipped sample and 43.8% for the sprayed sample at 20DAH. These two samples had a significant (P≤0.05) difference on their effect on weight loss at this day.

The dipped 1.5% xanthan/guar treated roots had a percentage weight loss of 38.6% while the sprayed root had 45.6% at 20DAH. There were significant (P≤0.05) differences between the two samples at this day.

The 2% xanthan guar treated roots had no significant (P>0.05) difference at 20DAH. The dipped sample had a percentage weight loss of 37.0% while the sprayed sample had 37.7%.

At 20DAH, the control root had a 50.2% weight loss and this was the highest as compared to the lowest recorded weight loss of 30.0% which was observed in the sample treated with 1.5% xanthan gum by dipping (Table 3). Generally, the treated cassava samples had no significant (P>0.05) differences on the weight loss during the entire storage duration.

Once the cassava roots have been harvested, their weight gradually reduces due to the loss of moisture and root respiratory activities [27]. This may have been the cause of the weight loss recorded in all the cassava root samples. The control cassava roots showed a higher weight loss as compared to the coated samples. This may have been due to the activity of the coating film which reduced the respiration rate hence reduced water lost from the roots. The weight lost increased with the storage duration as was recorded by Chen and Weil [28].

### 3.4. Dry Matter Content

The dry matter of the treated roots was determined and recorded for a period of twenty days on a two-day interval. There was an increase in the dry matter content of the treated roots as they approached 20DAH.

The dipped 1.5% xanthan treated root attained a dry matter content of 76.0% while the sprayed root sample had 72.5% at 20DAH which was significantly (P≤0.05) different. The samples treated with 1.5% xanthan/guar using dipping method had a content of 72.4% while the sprayed root had 71.7%. In addition, the 2% xanthan/guar dipped root had 64.6% while the sprayed root had 74.1% at 20DAH (Table 4).

All the treated roots had a significant (P≤0.05) difference at 20DAH as compared to the control root that had the highest dry matter content of 77.8%. Generally, there were significant (P≤0.05) differences between the differently treated roots. The initial dry matter content of the fresh cassava root was recorded as 56.1%, similar to the range of 10%-57% reported by Ebah-Djedji et al. [19]. They also stated that the dry matter content of cassava differs based on genotype and age at harvest. The increase in dry matter content of the cassava root is caused by the loss of moisture from the root surface due to various biochemical activities. This increases with the storage duration of the root [29]. In the present study, there was a general increase in the dry matter content of all the root samples as reported by Tumuhimbise et al. [30]. However, the control sample exhibited a higher dry matter content throughout the storage duration as compared to the coated root samples. This may be an indication of the effectiveness of the coating film formed in reducing the amount of moisture lost from the root samples. Depending on the storage conditions [31] the cassava root crops remain of acceptable eating quality even with increased dry matter content [32].

### 3.5. Total Phenolic Content

The total phenolic content was determined. This was recorded on the first day until 20DAH. There were no significant (P>0.05) differences amongst the various treatments.

The 1.5% xanthan dipped root sample had a decrease in its phenolic content to 8.4 mg/100g GAE while the spayed root sample had 9.1 mg/100g GAE at 20DAH. The 1.5% xanthan/guar treated root sample had 6.0 mg/100g GAE and 9.1 mg/100g GAE for the dipped and sprayed root sample respectively while the 2% xanthan/guar treated samples had 8.2 mg/100g GAE and 6.0 mg/100g GAE for the dipped and sprayed root samples, respectively. At 20DAH, the treated root samples had no significant (P>0.05) difference as compared to the control that had a total phenolic content of 7.7 mg/100g GAE. On coating using the various methods, there was a general gradual decline in the total phenolic content as it approached 20DAH (Table 5).

There was a decrease in the total phenolic content of the cassava root samples as was reported by [33]. This decrease continued with the time of storage. This may have been due to the increase in polyphenol oxidase with time hence the increased oxidation of the phenols and the eventual darkening of the flesh. The dark insoluble pigments that are formed during PPD are usually as a result of the oxidation of phenolic compounds in the cassava [34]. Enzymatic browning is directly correlated to the type and amount of the phenolic substrate. With PPD development, there is accumulation of phenolic secondary metabolites including scopoletin. These are the major phenolic compounds that may have led to the darkening of the cassava root flesh [35] with the storage duration. The coating process may have led to a delay in the oxidation of the phenols. There was a delay in the browning of the coated cassava as compared to the control. This may have been due to the inhibition of oxygen penetration to the cassava hence no formation of secondary metabolites.

### 3.6. Total Cyanide Content

The total cyanide content was analyzed and recoded for the 20 day storage duration. There was a decline of the total cyanide content from the first day to 20 DAH. The dipped 1.5% xanthan treated root attained a final cyanide content of 0.7ppm while the sprayed sample had 1.2ppm at 20 DAH. The 1.5% xanthan/guar treated roots had no significant difference on their effect on cyanide content at 20 DAH. The dipped root had 1.8ppm while the sprayed sample had 0.8ppm at 20 DAH. The dipped 2% xanthan/guar treated cassava root had a final cyanide content of 0.9ppm while the sprayed root had 1.4ppm at 20 DAH (Table 6). Generally, there was a decline in the total cyanide content of the cassava roots from the first day to 20 DAH. One of the major reasons why the cassava root crop is processed is to reduce the cyanide levels. Injuring of the cassava root crop during harvest triggers the contact of linamarin and linamarase enzyme which leads to an eventual production of hydrogen cyanide [36]. This process requires the presence of oxygen [37]. The coating process prevents oxygen access to the root hence it may have prevented the production of reactive oxygen species (ROS) that accelerate production of cyanide [25]. From the data obtained, the coated cassava root crops had an eventual low quantity of cyanide as compared to the control roots that were not coated and hence might have had a higher production of ROS which led to an increased cyanide content. Immediately after harvest, there was production of a high content of cyanide as was observed by Sowmyapriya et al. [36] and this reduced towards 20DAH. This may have been due to hydrolysis which may have broken down the cyanide [38].

### 3.7. Respiration Rate

The respiration rate of both the dip and spray treated sample was analyzed. Upon coating, the treated samples obtained two different peaks. The first peak occurred at the 2DAH while the second peak occurred at different days based on the treatment. The 1.5% xanthan treated root showed its first peak of 2.5 mg CO_2_/kg/h and 3.2 mg CO_2_/kg/h for the dipped and sprayed samples, respectively. The second peak was observed at 14DAH for the dipped sample and 12DAH for the sprayed sample at a respiration rate of 6.6 mg CO_2_/kg/h and 7.7 mg CO_2_/kg/h, respectively. The two samples had significant (P≤0.05) differences at 20DAH. The 1.5 xanthan/guar treated sample had their first peaks of 3.6 mg CO_2_/kg/h for the dipped sample while the sprayed sample had a respiration rate of 3.5 mg CO_2_/kg/h at 4DAH and 2DAH, respectively. The second peak was observed at 12DAH for sprayed sample while the dipped sample had its peak at 16DAH. The respiration rate for the samples was 5.7 mg CO_2_/kg/h for the sprayed sample while the dipped sample had 8.0 mg CO_2_/kg/h. The two samples had no significant (P>0.05) differences at 20DAH.

The 2% xanthan/guar treated roots attained their first peak at 2DAH for both the dipped and the sprayed roots. The dipped root had a respiration rate of 3.1 mg CO_2_/kg/h while the sprayed sample had a rate of 2.8 ml CO_2_/kg/h at the same day. The second peak of 6.5 mg CO_2_/kg/h was attained at the 12DAH for both the dipped and sprayed roots. The second peak was followed by a decline in the respiration rate towards zero as it approached 20DAH (Table 7). The control sample attained its second peak of 6.2 mg CO_2_/kg/h at 8DAH which was earlier than the other treated root samples. Generally, there was no significant difference between the two differently coated cassava roots.

The two peaks formed during the respiration process of the cassava root crop is due to wounding and biochemical changes for the 1st and 2nd peak respectively [39]. The increase in the cellular respiration of the cassava roots has been found to be a major contributor to the PPD [35]. This was observed in the present study as the control roots showed an increase in the respiration process as compared to the coated roots. As a result of the increased respiration rate, the PPD occurrence was higher in the control roots as compared to the coated roots. The increased respiration rate may lead to increased production of the reactive oxygen species which may have led to the enzymatic browning of the cassava root flesh [33].

### 3.8. Ethylene Production Rate

The ethylene production rate was determined from the first day to 20DAH. There were no significant differences between the differently coated roots. Upon coating, there was formation of one peak which was dependent on the efficiency of the coating solutions to reduce gaseous exchange between the cassava and the environment, which was followed by a decline.

The dipped 1.5% xanthan treated root attained a peak of 2.2 nl C_2_H_4_/g/h on 12DAH while the sprayed sample had 4.9 nl C_2_H_4_/g/h at 14DAH. The 1.5% xanthan/guar dipped sample had a peak of 2.2 nl C_2_H_4_/g/h at 14DAH while the sprayed sample had 2.8 nl C_2_H_4_/g/h on the same day. The sprayed 2% xanthan/guar treated root had its peak of 6.1 nl C_2_H_4_/g/h at 16DAH while the dipped sample did not attain a peak during the 20 day storage duration. At 20DAH, there was no significant difference in the various treated roots as the ethylene production rate range was very small-0.2 to 0.6 nl C_2_H_4_/g/h (Table 8). In general, there was no significant difference between the effects of the differently coated cassava root samples on the ethylene production rate.

Ethylene is one of the gases that affect various biochemical processes in various products. From the time of harvest, there was a slight increase in the ethylene production leading to formation of a peak and this might be attributed to the ethylene produced due to wounding [40, 41]. This peak formation varied with the different efficacy levels of the coating solutions. The early stages of PPD have been attributed to an increase in ethylene production which accelerates various enzymes [25]. This includes the peroxidase enzyme that has been found to oxidize phenols leading to production of secondary metabolites that lead to an eventual enzymatic browning on the cassava flesh. Ethylene production is induced by the production of ACC synthase and ACC oxidase enzymes which have been found to partly increase the activity of other enzymes which lead to the development of PPD. According to Saravanan [35], ethylene production has an intense effect on the development of PPD. This is contrary to the report by Liu [25] which stated the specific role that ethylene plays in PPD development is still unclear. The second peak formed for the various treatments was found to have no effect on PPD as was reported by HiRose (1984).

The thickness of the coating solution is crucial to the activity of the coat hence it should be well adjusted to suit its purpose. A very thick film may lead to anaerobic respiration hence the production of CO_2_, and off-flavors. The thickness of the coating directly correlates to the concentration, draining time and viscosity of the coating solution [12]. The concentrations used formed thin but effective films on the cassava.

## 4. Conclusion

All the coating solutions improved the postharvest life of cassava with respect to the control which was not coated. The application of edible coatings on the cassava root, extended the postharvest shelf life of the cassava with minimal alterations to its quality. The 1.5% xanthan/guar gum treated roots showed the best quality and had the longest shelf life extension of up to 20 days when stored at 25°C. Generally, no significant (P≤0.05) difference was recorded between the two different coating applications of dipping and spraying on the cassava root samples. No difference was noted on the colour changes, total cyanide content, total phenolic content, and firmness for all the treatments. However, the 1.5% differently coated xanthan roots had a significant difference on respiration rate and weight loss, while the 1.5% xanthan/guar gum coated roots had different effects on the ethylene production rate. The dipped and sprayed 2% xanthan/guar gum treated roots showed a significant difference in their effect on the dry matter content.

## Figures and Tables

**Table 1:** Changes in flesh firmness (Newtons) of cassava roots dipped and/or sprayed with different edible coating solution 20 days after harvest.

**Period of storage (Days)**	**TREATMENTS**								
	**Control**	**1.5**%** xanthan**	**1.5**%** xanthan**	**1.5**%** xanthan/guar**	**1.5**%** xanthan/guar**	**2**%** xanthan/guar**	**2**%** xanthan/guar**	**LSD**	**F**
		**Dipping**	**Spraying**	**Dipping**	**Spraying**	**Dipping**	**Spraying**	**(5**%** LEVEL)**	
**DAY 1**	61.8 ± 0.9^a^	61.8 ± 0.9^a^	61.8 ± 0.9^a^	61.8 ± 0.9^a^	61.8 ± 0.9^a^	61.8 ± 0.9^a^	61.8 ± 0.9^a^	2.7	1.000
**DAY 2**	71.4 ± 4.6^a^	65.7 ± 3.5^a^	62.2 ± 8.2^a^	66.5 ± 4.4^a^	73.1 ± 8.0^a^	63.8 ± 0.9^a^	69.4 ± 10.0^a^	19.4	0.871
**DAY 4**	76.8 ± 9.5^a^	70.2 ± 6.0^a^	64.8 ± 1.0^a^	68.1 ± 2.4^a^	76.2 ± 11.8^a^	69.9 ± 5.0^a^	81.5 ± 7.1^a^	21.4	0.667
**DAY 6**	80.5 ± 1.2^ab^	78.2 ± 1.2^ab^	69.5 ± 1.5^a^	76.9 ± 7.5^ab^	88.8 ± 6.8^b^	75.5 ± 0.5^ab^	84.0 ± 6.7^ab^	14.1	0.176
**DAY 8**	90.1 ± 4.5^cd^	79.4 ± 2.5^b^	71.2 ± 2.2^a^	95.8 ± 0.4^d^	89.2 ± 3.6^cd^	84.1 ± 1.5^bc^	88.0 ± 2.3^bcd^	8.2	<0.001
**DAY 10**	81.2 ± 9.3^bc^	92.5 ± 2.9^bc^	77.3 ± 3.4^b^	55.5 ± 1.1^a^	95.0 ± 1.4^c^	97.2 ± 1.9^c^	86.1 ± 8.8^bc^	14.1	0.176
**DAY 12**	54.8 ± 11.2^a^	91.4 ± 3.8^b^	95.6 ± 3.3^b^	50.8 ± 5.6^a^	96.0 ± 2.3^b^	66.3 ± 3.5^a^	61.7 ± 6.8^a^	18.0	<0.001
**DAY 14**	22.7 ± 0.9^a^	58.3 ± 4.0^c^	62.4 ± 2.5^c^	39.6 ± 9.5^b^	87.5 ± 3.3^d^	53.1 ± 1.5^bc^	51.4 ± 6.2^bc^	14.7	<0.001
**DAY 16**	15.9 ± 4.1^a^	46.7 ± 4.0^b^	49.3 ± 0.9^b^	49.5 ± 4.4^b^	78.2 ± 8.6^c^	52.4 ± 0.2^b^	42.6 ± 1.5^b^	13.0	<0.001
**DAY 18**	9.1 ± 0.6^a^	22.5 ± 5.4^ab^	36.0 ± 2.3^bc^	44.4 ± 6.1^cd^	54.2 ± 4.2^d^	48.7 ± 3.5^cd^	31.8 ± 10.3^bc^	16.5	<0.001
**DAY 20**	4.0 ± 0.7^a^	21.2 ± 3.3^b^	29.1 ± 2.6^bcde^	35.4 ± 2.2^ce^	46.1 ± 0.8^f^	24.5 ± 4.3^bcde^	24.3 ± 6.3^bcd^	10.3	<0.001

Values are means ± SE. Means with different superscript letters in a row are significantly (P≤0.05) different, n=3.

**Table 2:** Changes in colour (L*∗* values) of cassava roots dipped and/or sprayed with different edible coating solution 20 days after harvest.

**Period of storage (Days)**	**TREATMENTS**								
	**Control**	**1.5**%** xanthan**	**1.5**%** xanthan**	**1.5**%** xanthan/guar**	**1.5**%** xanthan/guar**	**2**%** xanthan/guar**	**2**%** xanthan/guar**	**LSD**	**F**
		**Dipping**	**Spraying**	**Dipping**	**Spraying**	**Dipping**	**Spraying**	**(5**%** LEVEL)**	
**DAY 1**	92.4 ± 0.3^a^	92.4 ± 0.3^a^	92.4 ± 0.3^a^	92.4 ± 0.3^a^	92.4 ± 0.3^a^	92.4 ± 0.3^a^	92.4 ± 0.3^a^	0.8	1.000
**DAY 2**	90.4 ± 1.1^b^	90.5 ± 0.7^b^	89.6 ± 1.2^ab^	90.5 ± 0.7^b^	87.6 ± 0.1^a^	91.0 ± 0.2^b^	90.9 ± 0.4^b^	2.2	0.054
**DAY 4**	86.5 ± 0.9^a^	89.8 ± 0.3^b^	89.3 ± 0.7^b^	90.5 ± 0.3^b^	90.6 ± 0.3^b^	89.9 ± 0.4^b^	90.2 ± 0.3^b^	1.5	<0.001
**DAY 6**	86.6 ± 1.1^a^	89.9 ± 0.2^b^	88.7 ± 0.1^b^	89.9 ± 0.2^b^	89.8 ± 0.5^b^	89.6 ± 0.2^b^	90.1 ± 0.4^b^	1.4	0.002
**DAY 8**	87.0 ± 1.1^a^	88.9 ± 0.8^ab^	88.4 ± 0.6^ab^	88.4 ± 0.3^ab^	89.7 ± 0.3^b^	89.2 ± 0.8^b^	89.7 ± 0.2^b^	1.9	0.122
**DAY 10**	82.2 ± 2.2^a^	89.0 ± 0.4^b^	87.9 ± 0.4^b^	86.7 ± 0.4^b^	89.6 ± 0.3^b^	88.0 ± 0.7^b^	88.0 ± 1.1^b^	1.4	0.002
**DAY 12**	79.3 ± 4.2^a^	88.3 ± 0.4^ab^	83.4 ± 4.0^ab^	86.8 ± 1.8^ab^	89.5 ± 0.3^b^	84.9 ± 3.4^ab^	88.0 ± 1.0^ab^	8.1	0.187
**DAY 14**	77.9 ± 1.6^a^	87.1 ± 0.1^b^	80.8 ± 5.7^ab^	84.9 ± 0.7^ab^	88.7 ± 0.3^b^	81.1 ± 1.6^ab^	85.7 ± 0.1^b^	7.1	0.054
**DAY 16**	77.3 ± 1.6^a^	86.0 ± 1.2^cd^	70.5 ± 5.1^a^	83.3 ± 0.3^bcd^	88.4 ± 0.2^d^	76.2 ± 5.1^ab^	85.6 ± 1.7^bcd^	8.8	0.006
**DAY 18**	73.6 ± 4.9^ab^	84.3 ± 1.0^ab^	70.3 ± 4.2^a^	82.4 ± 1.2^ab^	77.9 ± 8.0^ab^	75.5 ± 2.5^ab^	85.8 ± 2.0^b^	12.4	0.131
**DAY 20**	49.9 ± 7.0^a^	79.5 ± 3.3^cd^	65.9 ± 5.8^bc^	77.1 ± 0.9^cd^	60.2 ± 3.4^ab^	67.9 ± 4.5^bc^	83.2 ± 3.0^d^	13.3	0.001

Values are means ± SE. Means with different superscript letters in a row are significantly (P≤0.05) different, n=3.

**Table 3:** Changes in weight loss (percentage) of cassava roots dipped and/or sprayed with different edible coating solution 20 days after harvest.

**Period of storage (Days)**	**TREATMENTS**								
	**CONTROL**	**1.5**%** xanthan**	**1.5**%** xanthan**	**1.5**%** xanthan/guar**	**1.5**%** xanthan/guar**	**2**%** xanthan/guar**	**2**%** xanthan/guar**	**LSD**	**F**
		**Dipping**	**Spraying**	**Dipping**	**Spraying**	**Dipping**	**Spraying**	**(5**%** LEVEL)**	
**FRESH**	0	0	0	0	0	0	0	-	-
**DAY 2**	10.0 ± 0.4^cd^	6.0 ± 0.5^a^	11.3 ± 1.5^d^	9.3 ± 0.2^bcd^	9.3 ± 0.2^bcd^	7.3 ± 0.6^ab^	8.3 ± 0.2^bc^	2.0	0.001
**DAY 4**	15.9 ± 0.3^c^	13.1 ± 0.3^b^	16.6 ± 0.8^c^	13.7 ± 0.2^b^	12.8 ± 0.5^b^	10.3 ± 0.3^a^	11.2 ± 0.1^a^	1.2	<0.001
**DAY 6**	20.2 ± 0.2^d^	11.9 ± 0.2^a^	21.2 ± 0.8^d^	17.1 ± 0.1^c^	16.6 ± 0.2^c^	13.3 ± 0.2^b^	13.8 ± 0.4^b^	1.2	<0.001
**DAY 8**	25.4 ± 0.6^e^	15.1 ± 0.2^b^	29.2 ± 0.3^f^	20.9 ± 0.4^d^	21.2 ± 0.4^d^	17.1 ± 0.5^c^	13.1 ± 0.4^a^	1.3	<0.001
**DAY 10**	28.2 ± 0.2^d^	17.0 ± 0.5^a^	32.8 ± 1.4^e^	23.6 ± 0.4^bc^	24.5 ± 0.5^c^	24.4 ± 0.6^c^	21.7 ± 0.8^b^	1.2	<0.001
**DAY 12**	32.6 ± 0.5^e^	20.1 ± 0.2^a^	37.6 ± 1.0^f^	27.1 ± 0.5^c^	28.9 ± 0.5^d^	29.9 ± 0.4^d^	24.2 ± 0.2^b^	1.6	<0.001
**DAY 14**	35.9 ± 0.2^d^	23.0 ± 0.5^a^	39.6 ± 0.5^e^	31.0 ± 0.4^c^	35.0 ± 1.0^d^	31.2 ± 0.4^c^	29.0 ± 0.5^b^	1.6	<0.001
**DAY 16**	39.0 ± 0.5^c^	25.7 ± 0.6^a^	41.2 ± 0.7^d^	33.9 ± 0.4^b^	41.1 ± 0.1^d^	32.8 ± 1.2^b^	32.9 ± 0.3^b^	1.9	<0.001
**DAY 18**	44.6 ± 1.4^c^	27.6 ± 0.8^a^	42.1 ± 1.2^c^	35.7 ± 0.9^b^	43.4 ± 1.3^c^	35.7 ± 1.9^b^	34.5 ± 0.8^b^	3.8	<0.001
**DAY 20**	50.0 ± 0.1^d^	30.0 ± 1.7^a^	43.8 ± 1.4^c^	38.6 ± 1.7^b^	45.6 ± 0.9^cd^	37.0 ± 2.2^b^	37.7 ± 1.9^b^	4.7	<0.001

Values are means ± SE. Means with different superscript letters in a row are significantly (P≤0.05) different, n=3.

**Table 4:** Changes in dry matter content (percentage) of cassava roots dipped and/or sprayed with different edible coating solution 20 days after harvest.

**Period of storage (Days)**	**TREATMENTS**								
	**CONTROL**	**1.5**%** xanthan**	**1.5**%** xanthan**	**1.5**%** xanthan/guar**	**1.5**%** xanthan/guar**	**2**%** xanthan/guar**	**2**%** xanthan/guar**	**LSD (5**%** LEVEL)**	**F**
		**Dipping**	**Spraying**	**Dipping**	**Spraying**	**Dipping**	**Spraying**		
**FRESH**	56.1 ± 0.2^a^	56.1 ± 0.2^a^	56.1 ± 0.2^a^	56.1 ± 0.2^a^	56.1 ± 0.2^a^	56.1 ± 0.2^a^	56.1 ± 0.2^a^	0.6	1.000
**DAY 2**	62.9 ± 0.9^b^	57.4 ± 0.3^a^	55.9 ± 2.5^a^	57.7 ± 0.6^a^	54.6 ± 1.9^a^	53.1 ± 1.3^a^	56.2 ± 1.1^a^	4.3	0.006
**DAY 4**	64.6 ± 8.2^a^	60.2 ± 1.5^a^	57.9 ± 0.4^a^	58.9 ± 0.5^a^	55.0 ± 0.4^a^	55.9 ± 1.2^a^	60.5 ± 9.2^a^	14.3	0.821
**DAY 6**	67.9 ± 0.9^b^	62.7 ± 3.9^ab^	59.1 ± 0.6^ab^	61.3 ± 2.8^ab^	56.4 ± 0.4^ab^	55.9 ± 1.2^a^	61.6 ± 0.4^ab^	5.8	0.095
**DAY 8**	69.5 ± 1.0^b^	64.5 ± 1.6^ab^	59.7 ± 4.0^a^	61.5 ± 4.3^a^	57.7 ± 1.2^a^	56.6 ± 1.6^a^	61.9 ± 0.7^ab^	7.5	0.036
**DAY 10**	71.5 ± 2.4^d^	66.0 ± 1.6^c^	62.0 ± 0.6^bc^	62.1 ± 0.9^bc^	56.6 ± 0.5^a^	58.2 ± 1.3^ab^	63.0 ± 2.1^bc^	5.8	0.095
**DAY 12**	73.4 ± 2.0^c^	67.9 ± 5.2^abc^	62.8 ± 1.0^ab^	65.0 ± 1.4^abc^	58.3 ± 0.8^a^	62.2 ± 0.5^ab^	68.8 ± 0.7^bc^	9.3	0.047
**DAY 14**	73.4 ± 1.5^b^	67.4 ± 2.2^bc^	64.1 ± 0.9^abc^	67.9 ± 3.1^bcd^	58.8 ± 0.1^a^	62.6 ± 0.3^ab^	69.7 ± 2.9^cd^	5.6	0.001
**DAY 16**	73.4 ± 0.3^d^	73.8 ± 3.3^c^	70.5 ± 0.9^bc^	71.0 ± 1.5^bc^	67.2 ± 0.9^ab^	63.8 ± 1.3^a^	70.9 ± 0.6^bc^	5.2	0.012
**DAY 18**	74.3 ± 4.8^c^	74.8 ± 0.4^b^	71.6 ± 0.4^b^	71.3 ± 3.7^b^	71.1 ± 0.7^b^	63.9 ± 1.1^a^	72.3 ± 0.4^b^	4.9	0.008
**DAY 20**	77.8 ± 0.8^c^	76.0 ± 0.7^bc^	72.5 ± 2.8^b^	72.4 ± 0.7^b^	71.7 ± 0.9^b^	64.6 ± 1.5^a^	74.1 ± 0.6^bc^	4.1	<0.001

Values are means ± SE. Means with different superscript letters in a row are significantly (P≤0.05) different, n=3.

**Table 5:** Changes in total phenolic content (mg/100g GAE) of cassava roots dipped and/or sprayed with different edible coating solution 20 days after harvest.

**Period of storage (Days)**	**TREATMENTS**								
	**CONTROL**	**1.5**%** xanthan**	**1.5**%** xanthan**	**1.5**%** xanthan/guar**	**1.5**%** xanthan/guar**	**2**%** xanthan/guar**	**2**%** xanthan/guar**	**LSD**	**F**
		**Dipping**	**Spraying**	**Dipping**	**Spraying**	**Dipping**	**Spraying**	**(5**%** LEVEL)**	
**FRESH**	29.2 ± 2.1^a^	29.2 ± 2.1^a^	29.2 ± 2.1^a^	29.2 ± 2.1^a^	29.2 ± 2.1^a^	29.2 ± 2.1^a^	29.2 ± 2.1^a^	6.3	1.000
**DAY 2**	24.3 ± 1.9^ab^	17.9 ± 3.5^a^	27.7 ± 2.6^b^	18.5 ± 3.0^a^	27.7 ± 2.6^b^	23.4 ± 2.0^ab^	21.5 ± 2.1^ab^	7.9	0.091
**DAY 4**	19.9 ± 4.6^a^	16.7 ± 0.8^a^	22.5 ± 1.2^a^	21.6 ± 0.9^a^	22.5 ± 1.2^a^	20.2 ± 3.9^a^	19.6 ± 1.2^a^	7.4	0.668
**DAY 6**	17.0 ± 3.3^a^	14.5 ± 1.3^a^	18.1 ± 2.3^a^	16.2 ± 0.6^a^	18.1 ± 2.3^a^	17.8 ± 0.7^a^	14.5 ± 1.8^a^	6.0	0.693
**DAY 8**	15.2 ± 1.3^a^	13.6 ± 12^a^	15.1 ± 1.3^a^	15.2 ± 1.1^a^	15.1 ± 1.3^a^	14.5 ± 1.1^a^	13.1 ± 2.3^a^	4.3	0.89
**DAY 10**	14.5 ± 1.0^b^	12.6 ± 0.5^ab^	14.8 ± 1.0^b^	11.5 ± 0.3^a^	14.8 ± 1.0^b^	14.0 ± 1.0^ab^	13.1 ± 0.8^ab^	6.0	0.693
**DAY 12**	14.4 ± 2.2^a^	12.5 ± 0.2^a^	14.5 ± 1.4^a^	11.0 ± 1.1^a^	14.5 ± 1.4^a^	12.6 ± 0.7^a^	12.6 ± 0.3^a^	3.8	0.359
**DAY 14**	12.8 ± 0.9^a^	11.5 ± 0.6^a^	12.8 ± 0.2^a^	10.6 ± 2.2^a^	12.8 ± 0.2^a^	12.1 ± 0.8^a^	12.4 ± 1.1^a^	3.2	0.712
**DAY 16**	12.0 ± 0.9^a^	9.5 ± 3.8^a^	12.1 ± 1.0^a^	10.5 ± 1.2^a^	12.1 ± 1.0^a^	11.3 ± 1.5^a^	12.4 ± 1.7^a^	5.6	0.896
**DAY 18**	8.0 ± 1.0^a^	9.2 ± 0.9^ab^	9.4 ± 0.3^ab^	10.7 ± 0.5^b^	9.4 ± 0.3^ab^	9.5 ± 0.5^ab^	10.3 ± 1.4^ab^	2.4	0.365
**DAY 20**	7.7 ± 2.5^a^	8.4 ± 0.3^a^	9.1 ± 0.5^a^	6.0 ± 1.3^a^	9.1 ± 0.5^a^	8.2 ± 0.3^a^	6.0 ± 1.2^a^	3.6	0.361

Values are means ± SE. Means with different superscript letters in a row are significantly (P≤0.05) different, n=3.

**Table 6:** Changes in total cyanide content (ppm) of cassava roots dipped and/or sprayed with different edible coating solution 20 days after harvest.

**Period of storage (Days)**	**TREATMENTS**								
	**CONTROL**	**1.5**%** xanthan**	**1.5**%** xanthan**	**1.5**%** xanthan/guar**	**1.5**%** xanthan/guar**	**2**%** xanthan/guar**	**2**%** xanthan/guar**	**LSD**	**F**
		**Dipping**	**Spraying**	**Dipping**	**Spraying**	**Dipping**	**Spraying**	**(5**%** LEVEL)**	
**FRESH**	3.6 ± 0.4^a^	3.6 ± 0.4^a^	3.6 ± 0.4^a^	3.6 ± 0.4^a^	3.6 ± 0.4^a^	3.6 ± 0.4^a^	3.6 ± 0.4^a^	1.2	1.000
**DAY 2**	3.4 ± 0.6^a^	3.1 ± 0.3^a^	2.8 ± 0.3^a^	2.7 ± 0.1^a^	3.2 ± 0.2^a^	2.7 ± 0.2^a^	2.8 ± 7.1^a^	0.9	0.500
**DAY 4**	3.0 ± 0.5^a^	3.0 ± 0.2^a^	2.5 ± 3.2^a^	2.1 ± 0.5^a^	3.1 ± 0.5^a^	2.7 ± 0.6^a^	2.5 ± 4.3^a^	1.3	0.608
**DAY 6**	3.0 ± 0.3^a^	2.5 ± 0.3^a^	2.5 ± 4.4^a^	2.3 ± 0.2^a^	2.6 ± 0.3^a^	2.6 ± 0.2^a^	2.4 ± 7.4^a^	0.9	0.801
**DAY 8**	2.8 ± 0.4^a^	2.3 ± 0.3^a^	2.2 ± 3.1^a^	1.9 ± 0.2^a^	2.4 ± 0.1^a^	2.5 ± 0.6^a^	2.2 ± 4.1^a^	1.0	0.650
**DAY 10**	3.0 ± 0.3^a^	2.1 ± 0.1^a^	2.1 ± 2.2^a^	1.8 ± 0.4^a^	2.2 ± 0.1^a^	2.3 ± 0.4^a^	2.1 ± 0.2^a^	0.8	0.105
**DAY 12**	2.7 ± 0.1^a^	2.2 ± 0.3^a^	2.2 ± 4.2^a^	1.7 ± 0.2^a^	2.2 ± 0.5^a^	2.2 ± 0.1^a^	2.1 ± 0.1^a^	0.8	0.335
**DAY 14**	2.6 ± 0.2^a^	1.4 ± 0.5^a^	1.7 ± 0.2^a^	1.4 ± 0.1^a^	1.9 ± 1.0^a^	2.0 ± 0.4^a^	1.7 ± 0.1^a^	1.4	0.629
**DAY 16**	2.3 ± 0.1^a^	1.4 ± 0.1^a^	1.4 ± 0.3^a^	1.4 ± 0.1^a^	1.5 ± 0.1^a^	1.7 ± 0.3^a^	1.5 ± 0.1^a^	0.6	0.070
**DAY 18**	2.0 ± 0.5^a^	1.4 ± 0.4^a^	1.2 ± 0.2^a^	1.5 ± 0.2^a^	1.5 ± 0.1^a^	1.9 ± 0.1^a^	1.4 ± 0.1^a^	0.9	0.511
**DAY 20**	2.0 ± 0.2^a^	0.7 ± 0.1^a^	1.2 ± 0.3^a^	1.8 ± 0.1^a^	0.8 ± 0.1^a^	0.9 ± 0.3^a^	1.4 ± 0.1^a^	0.6	0.001

Values are means ± SE. Means with different superscript letters in a row are significantly (P≤0.05) different, n=3.

**Table 7:** Changes in total respiration rate (mg CO_2_/kg/h) of cassava roots dipped and/or sprayed with different edible coating solution 20 days after harvest.

**Period of storage (Days)**	**TREATMENTS**								
	**CONTROL**	**1.5**%** xanthan**	**1.5**%** xanthan**	**1.5**%** xanthan/guar**	**1.5**%** xanthan/guar**	**2**%** xanthan/guar**	**2**%** xanthan/guar**	**LSD**	**F**
		**Dipping**	**Spraying**	**Dipping**	**Spraying**	**Dipping**	**Spraying**	**(5**%** LEVEL)**	
**FRESH**	1.1 ± 0.1^cd^	0.7 ± 0.0^a^	1.1 ± 0.0^cd^	1.1 ± 0.0^d^	1.0 ± 0.0^c^	0.9 ± 0.0^b^	1.1 ± 0.1^cd^	0.1	<0.001
**DAY 2**	5.7 ± 0.0^ab^	2.5 ± 0.0^a^	3.2 ± 0.1^c^	2.5 ± 0.1^a^	3.5 ± 0.2^d^	3.1 ± 0.0^c^	2.8 ± 0.1^b^	0.2	<0.001
**DAY 4**	2.6 ± 0.0^ab^	3.9 ± 0.2^bc^	5.2 ± 1.6^c^	3.6 ± 0.1^bc^	1.1 ± 0.1^a^	2.9 ± 0.0^bc^	2.1 ± 0.0^ab^	1.8	0.006
**DAY 6**	3.4 ± 0.1^bcd^	1.3 ± 1.1^ab^	3.5 ± 0.3^e^	2.3 ± 0.1^cde^	1.9 ± 0.1^a^	3.2 ± 0.3^de^	2.9 ± 0.0^abc^	1.4	<0.001
**DAY 8**	6.2 ± 0.1^a^	3.09 ± 0.06^b^	5.38 ± 0.04^d^	4.47 ± 0.08^bc^	2.57 ± 0.23^c^	3.43 ± 0.22^c^	4.43 ± 0.03^a^	0.40	<0.001
**DAY 10**	3.04 ± 0.10^a^	4.6 ± 0.2^ab^	6.7 ± 0.2^c^	5.9 ± 0.0^bc^	3.6 ± 1.1^a^	4.3 ± 1.1^a^	4.2 ± 0.1^a^	1.4	<0.001
**DAY 12**	3.1 ± 0.1^a^	5.1 ± 0.0^a^	7.7 ± 0.2^ab^	6.1 ± 0.1^a^	5.7 ± 0.3^a^	6.5 ± 0.1^ab^	12.7 ± 5.3^b^	6.1	0.1
**DAY 14**	3.5 ± 0.1^a^	6.6 ± 0.1^bc^	7.1 ± 0.2^bc^	7.5 ± 0.1^c^	4.0 ± 1.2^a^	5.7 ± 0.2^b^	6.1 ± 0.2^bc^	1.5	<0.001
**DAY 16**	3.5 ± 0.1^a^	5.5 ± 0.0^bc^	7.3 ± 0.0^de^	8.0 ± 0.1^e^	3.1 ± 1.2^ab^	5.0 ± 0.0^bc^	5.4 ± 0.2^cd^	1.4	<0.001
**DAY 18**	4.0 ± 0.3^ab^	3.8 ± 1.5^a^	6.0 ± 0.6^bc^	6.5 ± 0.1^c^	4.9 ± 0.3^abc^	4.6 ± 0.2^abc^	4.5 ± 0.9^d^	2.1	0.002
**DAY 20**	3.7 ± 0.1^b^	0.5 ± 0.1^a^	3.8 ± 0.5^b^	3.6 ± 0.4^b^	3.2 ± 0.2^b^	3.7 ± 0.3^b^	3.4 ± 0.2^b^	0.9	<0.001

Values are means ± SE. Means with different superscript letters in a row are significantly (P≤0.05) different, n=3.

**Table 8:** Changes in total ethylene production rate (nl C_2_H_4_/g/h) of cassava roots dipped and/or sprayed with different edible coating solution 20 days after harvest.

**Period of storage (Days)**	**TREATMENTS**								
	**CONTROL**	**1.5**%** xanthan**	**1.5**%** xanthan**	**1.5**%** xanthan/guar**	**1.5**%** xanthan/guar**	**2**%** xanthan/guar**	**2**%** xanthan/guar**	**LSD**	**F**
		**Dipping**	**Spraying**	**Dipping**	**Spraying**	**Dipping**	**Spraying**	**(5**%** LEVEL)**	
**FRESH**	0.5 ± 0.0^a^	0.04 ± 0.0^a^	0.1 ± 0.0^a^	0.1 ± 0.1^a^	0.1 ± 0.0^a^	0.2 ± 0.2^a^	0.02 ± 0.0^a^	0.2	0.605
**DAY 2**	2.1 ± 0.0^a^	0.3 ± 0.0^a^	0.3 ± 0.0^a^	0.4 ± 0.1^a^	0.6 ± 0.1^a^	1.2 ± 1.2^a^	0.3 ± 0.0^a^	0.2	0.019
**DAY 4**	0.3 ± 0.0^c^	0.01 ± 0.0^a^	0.4 ± 0.0^d^	0.6 ± 0.0^e^	0.03 ± 0.0^a^	0.1 ± 0.0^b^	0.02 ± 0.0^a^	0.04	<0.001
**DAY 6**	0.1 ± 0.1^c^	0.1 ± 0.0^a^	0.3 ± 0.0^b^	0.02 ± 0.0^a^	0.3 ± 0.0^b^	0.1 ± 0.1^a^	0.1 ± 0.0^a^	0.1	<0.001
**DAY 8**	0.01 ± 0.0^a^	0.03 ± 0.0^a^	1.0 ± 0.1^c^	0.4 ± 0.3^ab^	1.5 ± 0.1^d^	1.6 ± 0.1^d^	0.7 ± 0.1^bc^	0.4	<0.001
**DAY 10**	0.4 ± 0.0^a^	0.9 ± 0.3^ab^	1.9 ± 0.9^b^	1.0 ± 0.3^ab^	1.2 ± 0.7^ab^	0.1 ± 0.0^ab^	1.0 ± 0.8^ab^	1.6	0.287
**DAY 12**	0.02 ± 0.1^a^	2.2 ± 0.1^b^	0.3 ± 0.0^a^	0.9 ± 0.5^a^	0.7 ± 0.1^a^	0.6 ± 0.0^a^	1.1 ± 0.5^a^	0.8	0.004
**DAY 14**	0.2 ± 0.0^a^	0.9 ± 0.2^bc^	2.9 ± 0.5^e^	2.2 ± 0.2^d^	2.8 ± 0.2^e^	0.5 ± 0.1^ab^	1.3 ± 0.0^c^	0.6	<0.001
**DAY 16**	12.4 ± 4.0^c^	0.8 ± 0.2^a^	4.9 ± 1.1^ab^	1.6 ± 0.3^ab^	0.7 ± 0.1^a^	0.7 ± 0.1^a^	6.1 ± 0.8^b^	4.9	0.001
**DAY 18**	0.2 ± 0.1^b^	0.3 ± 0.0^b^	0.1 ± 0.0^a^	0.1 ± 0.0^ab^	0.1 ± 0.0^a^	0.2 ± 0.0^ab^	0.6 ± 0.1^c^	0.1	<0.001
**DAY 20**	0.6 ± 0.0^ab^	0.3 ± 0.0^a^	1.0 ± 0.0^bc^	0.2 ± 0.1^a^	0.8 ± 0.4^ab^	0.3 ± 0.0^a^	1.5 ± 0.2^c^	0.6	0.003

Values are means ± SE. Means with different superscript letters in a row are significantly (P≤0.05) different, n=3.

## Data Availability

The data used to support the findings of this study are available from the corresponding author upon request.
